# Medicolegal litigation in general surgery: a comparison between England and the United States

**DOI:** 10.1186/s12961-025-01426-5

**Published:** 2025-12-06

**Authors:** Vivien Nebo, Michael G. Fadel, Liam Poynter, Hutan Ashrafian, Charles Bailey, Matyas Fehervari

**Affiliations:** 1 Department of General Surgery, Maidstone and Tunbridge Wells NHS Trust, Kent, United Kingdom; 2 Department of Surgery and Cancer, Imperial College London, London, United Kingdom; 3 University of Leeds Business School, Leeds, United Kingdom

## Abstract

**Background:**

Clinical negligence claims represent a significant financial and reputational burden for healthcare systems worldwide. While the United States is often perceived as having a highly litigious medical culture, comparative data between the United Kingdom and United States, especially in general surgery, are limited.

**Methods:**

Data on clinical negligence claims in general surgery were obtained through a Freedom of Information (FOI) request to NHS Resolution for England, covering financial years 2013/2014 to 2021/2022 and national databases from the United States between 2014 and 2022. Only successful claims with awarded damages were included. Population-adjusted annual means, total claim volumes and associated costs were calculated.

**Results:**

England recorded 5829 successful claims in general surgery over 9 years, with an estimated total cost of £873 million. The United States recorded 12 162 successful claims, which adjusted for population equated to 2043 claims, with an estimated adjusted cost of £563 million. England had three times more population-adjusted successful claims than the United States and nearly double the associated costs. The most common cause of successful litigation in England was “failure or delay in treatment”. Only 6.1% of successful claims were attributed to “operator error”.

**Conclusions:**

Despite the United States’ reputation for higher litigation, England had more successful, population-adjusted general surgery claims and costs over the study period. These findings highlight the importance of systemic, cultural and structural differences in how claims are handled and resolved in each healthcare system.

## Introduction

The outcome of surgery is generally improving in the United Kingdom and the United States. However, there has been an increase in the overall number of cases performed leading to higher number of litigations, which are not confined to the performance of the operations alone. For example, there are now approximately 12 000 medicolegal claims per year in England, resulting in a financial cost of more than £8 billion per year [1], with general surgery making up nearly 10% of all claims. These medicolegal claims are often associated with communication failure, inadequate clinical record keeping, poor training and lack of professional development, and system failure [2]

Litigation claims are usually resource-intensive, time-consuming and can be psychologically damaging to both the claimant and the defendant [3, 4]. In the context of the British National Health Service (NHS) as a free, point-of-use system, the financial and reputational effects of such claims have become a major concern, given the substantial legal costs involved [5]. However, the United States healthcare structure is a predominantly privatised insurance-based system. It has a long-standing reputation as being equipped to manage the risks and negative impact of excessive litigation claims and their associated costs [6]. In addition, the United States has centralised publicly available collaborative databases reviewing medical professional liability claims and lawsuits nationally, while the NHS collects its information through NHS Resolution (NHSR), whose registry is not available to the public.

Understanding the frequency of claims and associated costs is essential not only for financial reasons but also for maintaining the safety of surgical practice [7]. With general surgery resulting in a large proportion of all clinical claims [1, 8, 9], addressing the underlying causes of litigation could significantly reduce expenditure, which can then be redirected into improving patient care [10]. Secondarily, awareness of the most and least common types of successful litigation in England (type of injury) could provide a roadmap for where surgical practices must improve and focus to avoid reoccurring problems and litigation, highlighting methods to improve patient safety as well as protect surgeons in the future [11]. Furthermore, understanding these patterns could potentially have important implications for the broader healthcare infrastructure. Learning from medicolegal proceedings can guide policy revisions, shape training programs and frame clinical guidelines towards a more legally resilient surgical practice [12].

Previous articles have reviewed malpractice claims within different healthcare systems, to identify factors that might result in a higher future risk of claims [13–19]. However, none to date have compared the incidence and costs of claims between different higher-income countries. We therefore reviewed medicolegal data in the United Kingdom, specifically England, and the United States, to suggest strategies for improvement and risk mitigation.

## Methods

Medicolegal data from claims made against NHS General Surgical Departments in England were formally requested from NHSR under the Freedom of Information Act over the financial years 2013/2014 to 2021/2022, encompassing a 9-year period. Retrospective review of the following clinical claims was performed:

1. Number and cost of general surgery clinical claims closed [or settled as a Periodical Payment Order (PPO)] between financial years 2013/2014 and 2021/2022, with damages (includes the damages paid to date for any claims settled on a PPO basis).
2. Number and cost of general surgery clinical claims closed between financial years 2013/2014 and 2021/2022 with nil damages paid.
3. Analysis of primary injuries with five or more general surgery clinical claims closed (or settled as a PPO) between financial years 2013/2014 and 2021/2022 with damages (includes the damages paid to date for any claims settled on a PPO basis).
4. Analysis of primary causes with five or more general surgery clinical claims closed (or settled as a PPO) between financial years 2013/2014 and 2021/2022 with damages (includes the damages paid to date for any claims settled on a PPO basis).
5. Number and cost of general surgery clinical claims closed (or settled as a PPO) between financial years 2013/2014 and 2021/2022 with damages (includes the damages paid to date for any claims settled on a PPO basis). Broken down by litigation status.
6. Number and cost of general surgery clinical claims that went to trial closed (or settled as a PPO) between financial years 2013/2014 and 2021/2022 with damages (includes the damages paid to date for any claims settled on a PPO basis).

A successful general surgery clinical claim was therefore defined as any claim where damages (not simply legal costs) were awarded upon closure of the claim, whether this was via litigation or an out-of-court settlement.

The data are correct up to August 2023, and the information only covers England and not the rest of the United Kingdom. General surgery encompasses all subspecialties traditionally included in general surgery but not including vascular surgery. Detailed information regarding individual case details is suppressed to not provide information which can identify claimants or members of staff. This is particularly relevant to areas with low case numbers where disclosure to a member of the public would contravene one or more data protection principles as set out in Article 5 of the General Data Protection Regulation. Where fewer than five claims are made in each category, it is deemed that this information is sensitive personal data, where disclosure could potentially cause damage or distress to those involved, and so a “#” symbol is placed in the relevant field. Aggregate totals pertaining to higher-level fields remain; however, where data interpretation remains possible, some total values may be approximated to prevent deduction of masked values through reverse calculation. PPO are an agreement between parties to pay an initial lump sum and regular future payments covering the injured claimant’s ongoing care needs, usually for life.

To review the medicolegal landscape across general surgery in the United States, the National Practitioner Data Bank (NPDB) was reviewed, which yields confidential data across all payments reported against all health practitioners. We reviewed the data across the same timeframe between 2014 and 2022. The NPDB does not subcategorise payment reports by speciality and so an estimate was obtained to determine the proportion of claims observed in general surgery. In a previous study by Studdert et al. examining the medicolegal landscape in the United States, an estimate of 12% of all cases were found to be from doctors in general surgery [13]. This estimate was obtained from the total number of active physicians in the United States, drawn from the American Medical Association, according to speciality and year [20]. Population-adjusted case numbers and claim values were used to proportionally compare the United States and England. At a current population of over 333 million in the United States against just under 56 million in England, a ratio of ~5.95 was applied to assess caseload and expenditure across the two nations.

Data were entered into Microsoft Excel (Microsoft Corp., Redmond, WA, United States) and analysed using IBM SPSS Statistics version 28 (IBM Corp., Armonk, NY, United States). Descriptive statistics were used to calculate annual means, standard deviations, and ranges for claim numbers and awarded damages. Population adjustment was performed using national census data for the relevant years, enabling direct comparison between the two healthcare systems. Currency conversion was applied using a fixed exchange rate of 1 USD = 0.79 British pound sterling (GBP) on the basis of average market value at the time of analysis. Figures were produced in Excel and SPSS and included bar graphs and line charts illustrating annual claim volumes and total costs, both raw and population-adjusted. No inferential statistical tests were applied, as this was a descriptive, comparative study of aggregated national data.

### NHS England litigation process

**Fig. 1:**
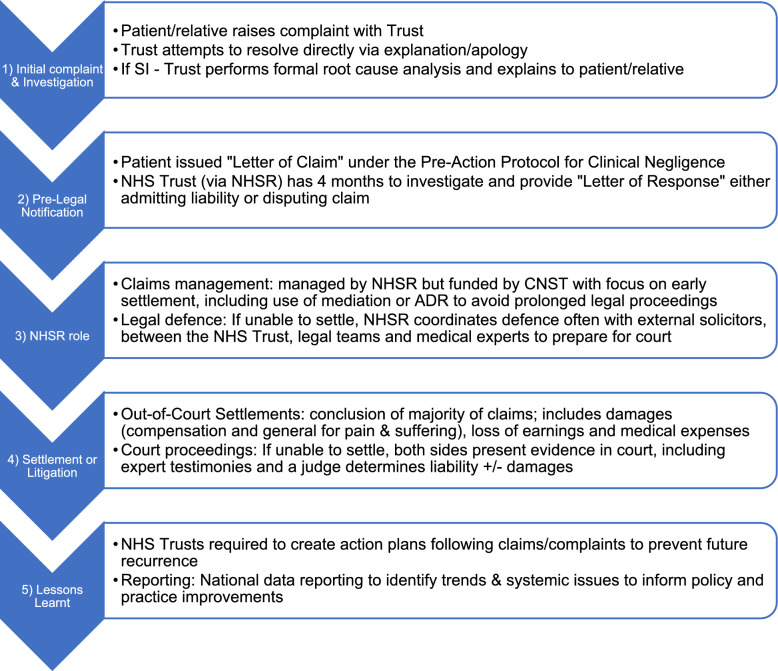
Summary of the NHS litigation process in England/United Kingdom [21, 22]. ADR, alternative dispute resolution; CNST, Clinical Negligence Scheme for Trusts; NHSR, NHS Resolution; SI, serious incident

**Fig. 2:**
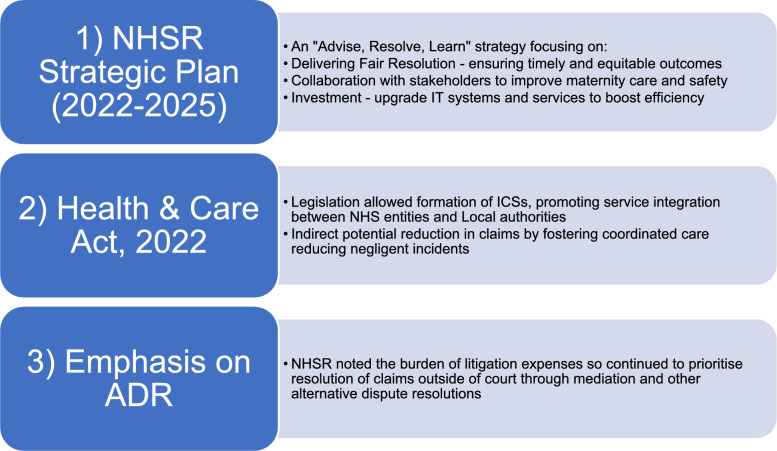
Key strategies implemented by the NHS to address rising financial burden associated with clinical negligence claims [23, 24]. ADR, alternative dispute resolution; ICS, Integrated Care System; NHSR, NHS Resolution

To date, the core framework for management of medicolegal claims has remained consistent in the NHS; however, in 2022, three key strategic enhancements were implemented, aiming to increase efficiency, foster collaboration, and address the rising financial burden associated with clinical negligence claims [23] (Figs. 1 and 2).

Despite all the above efforts to manage claims more effectively, NHSR clinical negligence claims costs have increased yearly to date, intensifying calls for further legal reforms to ensure the sustainability of compensation systems. [25]

### United States healthcare system litigation process

**Fig. 3:**
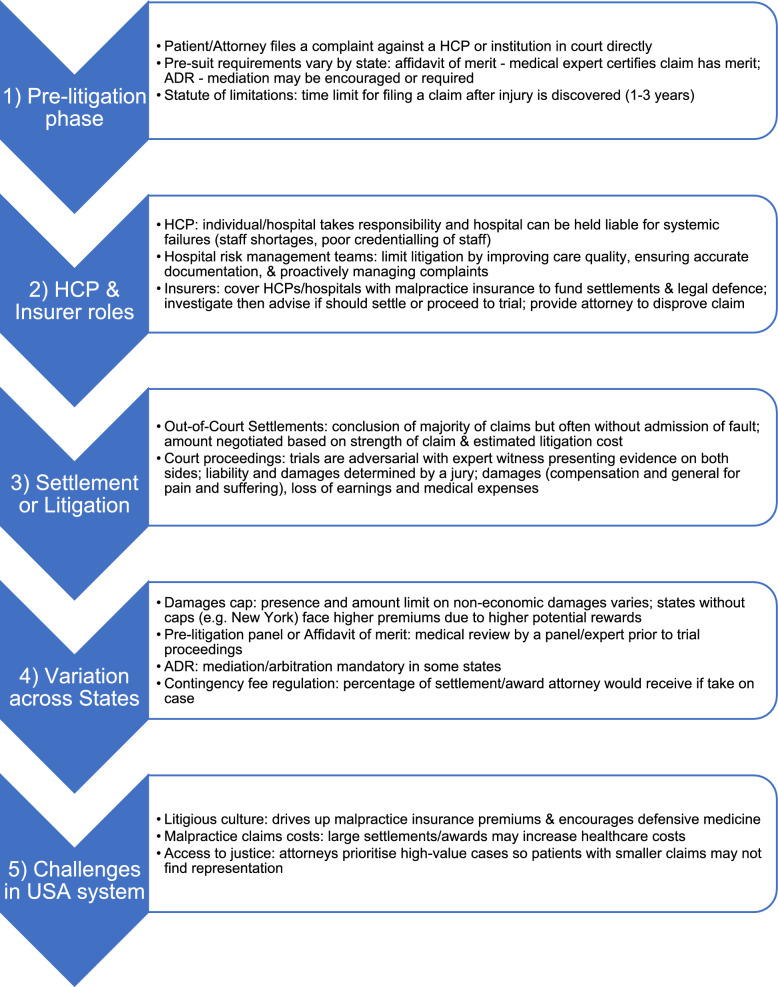
Summary of the litigation process in the United States [26–28]. ADR, alternative dispute resolution; HCP, healthcare practitioner

The United States healthcare litigation process varies significantly, owing to the private healthcare system, state-based legal frameworks and the litigious nature of their society [26] compared with the United Kingdom (Fig. 3).

## Results

### Comparison of claims in England and the United States

**Fig. 4:**
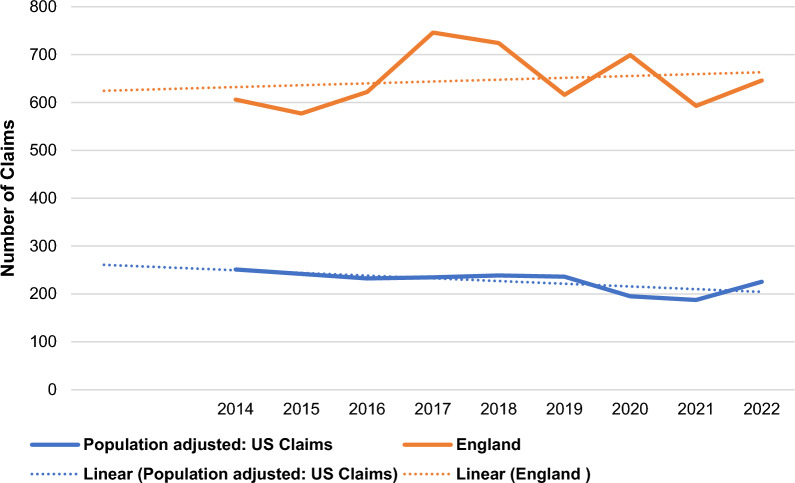
Annual claim numbers, adjusted by population size, in England and the United States

**Fig. 5:**
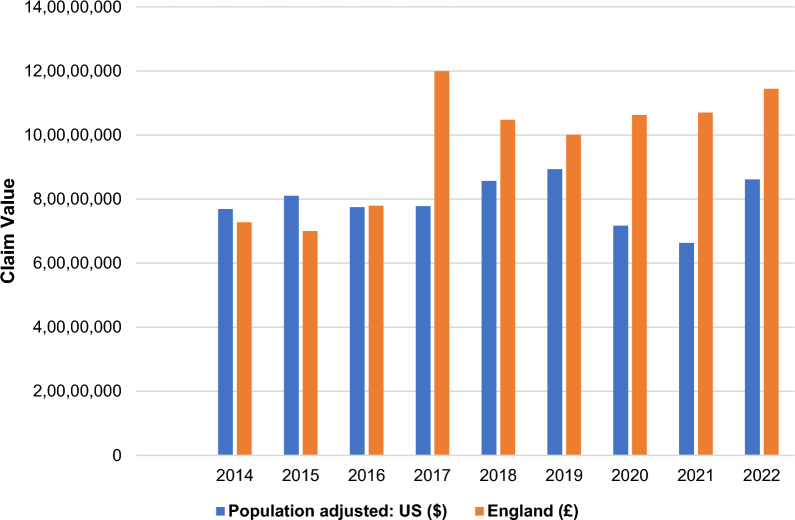
Annual claim value, in pounds and dollars, adjusted by population size, in England and the United States

In England, a total of 9742 claims were made between financial years 2013/2014 and 2021/2022 against general surgery departments (annual mean, 1082; range, 894–1505). Of these, 5829 claims were successful and awarded damages (annual mean, 648; range, 577–746) (Fig. 4). The total cost of closed successful clinical claims over the 9-year period in England was approximately £873 000 000 (mean, £97 000 000; range, £73 000 000–120 000 000 per year) (Fig. 5).

In the United States, a total of 12 162 estimated claims made between 2014 and 2022 against general surgery were successful and awarded damages (annual mean, 1351; range, 1116–1495), which, when population-adjusted, amounted to a total of 2043 claims that were successful and awarded damages (annual mean, 227; range, 187–251). The estimated total cost of closed successful clinical claims over the 9-year period in the United States was approximately US $4 241 000 000 (mean, $471 000 000; range, $395 000 000–532 000 000 per year), which, when population-adjusted, was approximately $712 000 000 (mean, $79 000 000; range, $66 000 000–89 000 000 per year). Using the current exchange ratio for dollars to pounds of 0.79 (at the time of analysis) [29], the population-adjusted total cost of closed successful claims over the 9-year period in the United States was approximately £563 000 000 (mean, £63 000 000; range, £52 000 000–71 000 000 per year).

### Successful claims according to types of injury and cause of injury in England

Analysis of patterns of primary injuries sustained by general surgical patients resulting in successful clinical claims in England revealed “additional/unnecessary operations” as both the most frequent successful claim and the highest cost between 2013/2014 and 2021/2022, accounting for 19.5% of overall claims and 15.9% of expenditure (Supplementary Table S1). “Unnecessary pain”, “fatality” and “bowel damage/dysfunction” accounted for 16.2%, 10.6% and 8% of injury types incurred in successful clinical claims, respectively, corresponding to 8.8%, 11.96% and 14.8% of total expenditure.

Only 6.1% of successful claims were due to “operator error” – the fifth highest legally determined cause of injury, amounting to only 5.8% of total damages and legal costs paid (Supplementary Table S2). The number one cause for injury sustained by patients in the 9-year period was “failure/delay in treatment” (19.0% of total claims; 20.8% of total costs), followed by “intraoperative problems” (12.97% of total claims; 17.3% of total expenditure); “failure/delay diagnosis” (12.7% of total claims; 16.6% of total expenditure); “inappropriate treatment” (9.4% of total claims; 8% of total expenditure), “inadequate nursing” (4.5% of total claims; 2.1% of total expenditure), and finally by “failure to warn – informed consent” (4.3% of total claims; 4.7% of total expenditure). The average total cost of damages and legal fees was £149 810 per successful claim.

## Discussion

This study demonstrated that there were more successful claims and associated awarded damages against general surgery departments in England than in the United States, after adjusting for population and exchange rate differences. There were approximately three times more population-adjusted successful claims in England, and almost twice as much in damages awarded over the 9-year period. These findings were somewhat unexpected considering that, in contrast to the NHS, the United States healthcare structure is a predominantly privatised insurance-based system which is well-versed with the risks and negative impact of excessive litigation claims and their associated costs [30], and anecdotally, litigation was thought to be higher. This false perception may be owing to the key differences between civil cases in England and the United States; the former is assessed by a single judge, while the latter uses jury trials [31], which can sometimes result in unpredictable and dramatic high-damages cases.

As a publicly funded centralised system, litigation against the NHS is managed by the government body NHSR [25]. Its 2024 annual report states “a record of 81% of claims” were settled prior to legal court proceedings, which they theorise reduces overall costs by avoiding court proceedings. However, it could be argued that without the strict adjudication from a judge, this strategy might increase the number of successful claims, leading to higher overall damages costs. Our study noted that 5804 of 5829 successful claims (99.6%) were closed without going to trial, resulting in over £850 million legal fees and damages payments, albeit avoiding court costs. In total, 25 successful claims went to trial, amounting to just over £22 million in damages and legal costs in total. In addition, the streamlined consolidation of majority of the British population under the single NHS system may provide more accurate reporting of the number and costs of claims than that of the United States’ system, which is fragmented across many private insurers and could be causing underreporting. [32]

Our data showed that NHSR spent almost £69 million on legal costs for those with successful claims between financial years 2013/2014 and 2021/2022 and over £12 million on the legal costs of those with unsuccessful claims. In England, NHSR covers the legal costs of both successful and many unsuccessful claimants [25], while the burden of legal costs in the United States is on the patient [31], which may limit access to justice to those without the financial means to cover upfront legal costs and attorney fees. The prevalence of “no-win-no-fee" solicitor services in the United Kingdom may also contribute to reducing the barriers for patients to pursue claims, while in the United States both parties are expected to bear their own costs, unless specifically stipulated [31]. The presence of contingency fees in the United States may encourage attorneys to prioritise high-value cases, so smaller or borderline cases may not find representation, while in the United Kingdom, all claims are investigated. Of the represented cases that are awarded, United States settlements only include high punitive damages in rare cases [6]; many private insurers exclude payout for broader claims that the NHS covers, such as rehabilitation, future earnings, or life-long healthcare costs [33], and some US states have a cap on noneconomic damages such as pain and suffering [34], unlike in the United Kingdom.

The NHS constitution pledges to learn from any mistake, fostering a culture of openness key to maintaining public trust [35, 36]. This is associated with reduced mortality [37], and it could be argued that transparent compensation of harmed patients strengthens the societal perception of its commitment to fairness and patient welfare. For example, the high rates of successful maternity and birth-related claims have triggered systemic reviews and reforms that are ongoing, in an endeavour to improve patient safety in this critical area [38, 39]. However, this admirable patient-focused care that the NHS strives to provide could be driving up the number of successful claims. Conversely, the high awareness of litigation in the United States may lead to a fear of reporting errors that could drive systemic change, owing to the risk of being named in possible future lawsuits. Fear of litigation can lead to unnecessary investigations or treatments, known as “defensive medicine”, or even avoiding treating patients with certain medical conditions associated with higher rates of litigation, which may limit or stop the practice of some doctors altogether. [6, 30]

The NHS has its own limitations and systemic issues. Workforce shortages have posed a significant challenge within the NHS, with high staff vacancies being exacerbated by the increasing migration of staff overseas seeking improved work–life balance and remuneration for hours worked [40]. Lord Darzi’s recent report affirms that many of the issues have been exacerbated by the Coronavirus-19 pandemic and the subsequent significant rise in inflation [41, 42]. Productivity has continued to decline, primarily owing to poor flow through hospitals, a shortage of capital to increase appointments and procedures, and underinvestment in community and social care [41]. The impact of these systemic issues on patient care cannot be understated, inevitably resulting in increased strain on the system and a higher potential for error.

The healthcare system in the United States receives a lot of private investment, which some conclude leads to reduced financial constraints, motivates innovation and higher quality healthcare [43, 44]. This may result in more robust consent, particularly in an environment adept at malpractice lawsuits, which necessitates a strong legal standing. However, many more argue that the increasing costs of healthcare negatively impact the quality of care and limit access to only the affluent who can afford the cost but may not be most in need of care [6, 45]. This disproportionally affects patients from lower socioeconomic backgrounds and deprived areas [46]. Some other negative effects of privatisation and increased healthcare costs include restriction of necessary treatments by insurers, causing healthcare delays, financial jeopardy for patients too unwell to work (especially if insurance is tied to their employment), a focus upon specialist treatment of disease rather than prevention, stifling of investment in cost–effective innovation that could improve care but may not increase financial returns to medical companies, and fragmented care due to a lack of integration of various healthcare services. [6, 30, 46]

While there are numerous areas requiring improvement in the NHS, adopting a privatised healthcare system such as the United States may not be the solution. The Commonwealth Fund recently performed an in-depth analysis of the performance of healthcare systems of 10 high-income countries, titled “Mirror, Mirror 2024” [47]. The United Kingdom ranked third overall behind Australia and the Netherlands, while the United States healthcare system was the lowest performing by a significant margin. Yet, there remain some key improvements the NHS could make, which the redistribution of costs from litigation to healthcare services could aid.

### Learning from types of litigation

Identifying patterns in common litigation types can help proactively mitigate future risk, alleviate financial burden and reduce preventable complications. A recent retrospective review of litigation trends in general surgery identified “additional/unnecessary operation(s)” and “unnecessary pain” as the most common primary injuries associated with successful claims [1]. Our study found that 35.65% of claims and 24.76% of expenditure, amounting to over £213 million in damages and legal fees, were owing to these injuries. Other primary injuries sustained included unnecessary pain, fatality, bowel damage/dysfunction and bile duct damage. It could be argued that providing patients with detailed informed consent for their procedure could have mitigated or even prevented claims of this nature. Throughout the consent process, risks and relative success of the procedure should be stated clearly at an appropriate level for the patient’s understanding and alternative management options, including conservative management should be discussed to manage the patients expectations and prepare them for potential surgical complications [48]. Improvements to reduce litigation claims, in addition to this, need to focus on clear documentation, multidisciplinary team planning, development of regular internal claims learning processes and more accurate detection of postoperative complications.

Although our data do not clearly establish whether higher litigation claim rates are predictive of poorer clinical outcomes, it is notable that 10.5% of successful claims and 11.8% of total payouts, exceeding £103 million, were associated with patient fatalities. Further research exploring the relationship between claim frequency and morbidity or mortality outcomes could yield valuable insights. Should a significant correlation be identified, the implementation of regular joint litigation morbidity and mortality (M&M) meetings could review cases, extract key learning points and ultimately reduce future surgical litigation claims and expenses, while most importantly enhancing patient safety.

The widespread application of the World Health Organization Surgical Safety Checklist (WHOSSC) has significantly reduced rates of wrong-site, wrong-patient and wrong-procedure occurrences [49]. Its use reduced mortality by almost 50% and complications by over 60% in a global collaborative prospective study involving eight major cities across the world [50]. This is exemplified by our data; only 0.7% of successful claims and 0.35% of total litigation expenditure were determined to be caused by “wrong-site surgery” or “incorrect injection site”. De Vries et al. developed a checklist, the Surgical Patient Safety System (SURPASS), based on similar principles to the WHOSSC, but spanning the entire surgical pathway, focusing on admission, discharge and patient transfer points, which is now widely used in the Netherlands [51]. Items include postoperative anaesthetic medication checks, surgical confirmation of drain and diet instructions, preoperative ward doctor review of the patient and blood results, and confirmation of a follow-up plan prior to discharge. Application of a similar checklist within the NHS could streamline many perioperative checks and significantly reduce litigation claims due to failure to “perform tests”, “follow-up”, “act upon abnormal test results” and “medication errors”, amongst others. These four categories alone accounted for 317 successful claims, totalling almost £40 million in litigation expenses (Supplementary Table S2). However, use of a similar checklist would need to be weighed against the ability of surgical teams to adhere to such detailed checklists in the context of an already overwhelmed NHS climate, plus the associated implementation costs.

In the context of surgical litigation, integrating artificial intelligence (AI) with national audit data could enable the early detection of rising complication rates, providing digital alerts before issues escalate into formal complaints [52]. Identifying certain patterns in patient care, including the type of disease, frequency of patient and family communications, and perhaps continuous recording of in-hospital satisfaction, could proactively identify potentially litigious cases early on. Such a system would closely align with NHSR’s objective to manage and resolve complaints swiftly [25] while delivering real-time feedback to clinical teams, ultimately contributing to a reduction in complications and a measurable improvement in patient safety. Despite its vast potential, the integration of AI into healthcare has raised significant concerns. These include the risk of false negatives or positives, which could lead to missed diagnoses or unnecessary interventions, respectively; the possibility of conscious or unconscious bias, potentially exacerbating existing health inequalities; and a lack of transparency in the development and deployment of AI systems, raising fears around patient data privacy and security. [53, 54]

Lastly, while the focus should remain upon preventing patient complications and the resulting litigation claims, attention should be given to the secondary victim: the accused healthcare professional(s) after an incident occurs or a claim is raised [55]. The loss of professional reputation alone can have far-reaching consequences, including diminished social standing and ostracism, as well as self-imposed isolation [56]. The stress of litigation can erode confidence in one’s abilities and skills, instilling fear and self-doubt, leading to adoption of defensive medicine practices [57]. Madan et al. proposed a number of strategies that should be implemented by individual practitioners, healthcare institutions, and governing bodies and systems to provide structured support for healthcare professionals following adverse events [56]. These include prevention, through robust medical education and ongoing professional development and mitigating factors such as burnout and stress. Support during the litigation process is equally vital, encompassing peer debriefing, access to counselling and timely medicolegal advice. Strengthening local dispute resolution mechanisms could help address issues early, preventing escalation into formal legal action. Institutional debriefing should aim to shift the culture around litigation from one of blame to one focused on reflection, learning and systemic improvement [56].

### Limitations

Several factors may have limited the accuracy of direct comparison of data between England and the United States. An estimate of claims attributable to general surgery in the United States was made, as the NPDB does not subcategorize payment reports by speciality. The NHSR PPO policy means that some damages awarded that year may be for a previously settled claim for ongoing patient care needs. This may have skewed the estimated costs of successful claims. As discussed above, the English and American healthcare and litigious systems vary significantly, which may limit the accuracy of comparing outcomes.

## Conclusions

Over a 9-year period, there were more successful claims and associated awarded damages against general surgery departments in England than in the United States, when population and exchange rate differences were adjusted for. This could be due to several factors, including variations in access to the pursuit of a claim or differences in judicial systems and healthcare system cultures. Patterns in types of successful litigation could provide a roadmap of learning for surgeons and within General Surgical departments across the country. Surgical practices should focus on reform to engage clinicians in thorough informed consent, clear documentation and development of regular internal claims learning processes to reduce preventable complications and avoid recurring litigation. Addressing these issues is crucial to mitigating risk, improving patient safety, reducing the financial burden of litigation and protecting the surgeons of the future.

## Data Availability

No datasets were generated or analysed during the current study.
